# Evaluating the Clinical- and Cost-Effectiveness of Cochlear Implant Sound Processor Upgrades in Older Adults: Outcomes from a Large Australian Multicenter Study

**DOI:** 10.3390/jcm14113765

**Published:** 2025-05-28

**Authors:** Paola Vittoria Incerti, Jermy Pang, Jason Gavrilis, Vicky W. Zhang, Jessica Tsiolkas, Rajan Sharma, Elizabeth Seil, Antonio Ahumada-Canale, Bonny Parkinson, Padraig Thomas Kitterick

**Affiliations:** 1 National Acoustic Laboratories, Macquarie University, Sydney, NSW 2109, Australia; jermy.pang@nal.gov.au (J.P.); jason.gavrilis@nal.gov.au (J.G.); vicky.zhang@nal.gov.au (V.W.Z.); jessica.tsiolkas@nal.gov.au (J.T.); padraig.kitterick@nal.gov.au (P.T.K.); 2 Macquarie University Centre for the Health Economy, Macquarie University, Sydney, NSW 2109, Australia; rajan.sharma@mq.edu.au (R.S.); antonio.ahumadacanale@uts.edu.au (A.A.-C.); bonny.parkinson@mq.edu.au (B.P.)

**Keywords:** cochlear implants, obsolescence, sound processor upgrade, cost-effectiveness, clinical effectiveness, older adults

## Abstract

**Background:** Many older Australian adults with cochlear implants (CI) lack funding for replacement sound processors, risking complete device failure and reduced quality of life. The need for replacement CI devices for individuals with obsolete sound processors and no access to funding poses an increasing public health challenge in Australia and worldwide. We aimed to investigate the clinical and cost-effectiveness of upgrading obsolete CI sound processors in older adults. **Methods:** Alongside an Australian Government-funded upgrade program, a prospective, mixed-methodology design study was undertaken. Participants were aged 65 and over, with obsolete Cochlear™ sound processors and no funding for replacements. This study compared speech perception in noise, as well as self-reported outcome measures, including cognition, listening effort, fatigue, device benefit, mental well-being, participation, empowerment and user experiences, between upgraded and obsolete hearing aid processors. The economic impact of the upgrade was evaluated using two state-transition microsimulation models of adults using CIs. **Results:** The multi-site study ran from 20 May 2021 to 21 April 2023, with recruitment from June 2021 to May 2022. A total of 340 Cochlear™ sound processors were upgraded in 304 adults. The adults’ mean age was 77.4 years (SD 6.6), and 48.5% were female. Hearing loss onset occurred on average at 30 years (SD 21.0), with 12 years (SD 6.2) of CI use. The outcomes show significant improvements in speech understanding in noise and reduced communication difficulties, self-reported listening effort and fatigue. Semi-structured interviews have revealed that upgrades alleviated the anxiety and fear of sudden processor failure. Health economic analysis found that the cost-effectiveness of upgrades stemmed from preventing device failures, rather than from access to newer technology features. **Conclusions:** Our study identified significant clinical and self-reported benefits from upgrading Cochlear™ sound processors. Economic value came from avoiding scenarios where a total failure of device renders its user unable to access sound. The evidence gathered can be used to inform policy on CI processor upgrades for older adults.

## 1. Introduction

Research shows that people with untreated hearing loss experience reduced social participation, anxiety, depression, and low health status [1,2,3,4]. In older adults, there is a clear association between hearing loss, cognitive decline and dementia [5], whereby there is a five-fold increased risk of dementia in those with severe hearing loss [6] and a greater risk of injury due to falls and other accidents [7], and they are far more likely to be admitted to hospital for longer period of time than their hearing peers [8].

Cochlear implants (CI) are a cost-effective, lifelong intervention for individuals with severe to profound sensorineural hearing loss [9,10]. The CI system consists of two main components: a surgically implanted device and an externally worn sound processor. The sound processor captures sound via a microphone, processes it, and transmits it to the internal implant, which electrically stimulates the auditory nerve in the cochlea.

Recipients of CIs commit to lifelong use, particularly of the external sound processor, which requires periodic upgrades to incorporate advancing technology [11]. These upgrades necessitate clinic visits but not surgery. Many older Australian adults currently use obsolete sound processors, and do not have access to public or private health insurance funding for replacement sound processors. As obsolete technology is no longer manufactured, access to spare parts become increasingly difficult, and over time, services and repairs also cease to exist. This leads to complete device failure, which translates to an inability to use their internal implant, the complete loss of sound access and severe restrictions on daily activities. Given the growing evidence on the impact of untreated hearing loss and the potential impact on quality of life, it is critical that every effort is made to ensure that those with CIs have fully functioning devices.

Outcome measures for CI sound processor upgrades in users typically focus on speech intelligibility tests in quiet and noisy conditions [12]. These tests are valuable, patient-centered measures that address meaningful aspects of hearing loss as identified by adults who are deaf or hard of hearing, and clinicians [13] can offer new insights into the benefits of upgraded processors. There is a lack of literature relating to the costs and benefits associated with health, well-being and societal contributions [14]. This gap includes insufficient research on financial costs associated with CI sound processor upgrades, and research regarding long-term outcome measurement following these upgrades. Consequently, there is limited evidence to guide health policy development regarding CI sound processor upgrades.

This study aims to evaluate the short- and long-term effects and lived experience of upgrading obsolete or near-obsolete sound processors, alongside a cost-effectiveness analysis for adults aged 65 years and older who use CIs. The primary objectives of the study were to compare speech perception in noise between upgraded and obsolete sound processors after 4 weeks and 6 months, and to gather insights through pre- and post-upgrade measures of self-reported benefit and lived experiences. Additionally, the study conducts a health economic evaluation of the upgrade program from the perspective of the Australian health system.

## 2. Materials and Methods

### 2.1. Study Design and Participants

A multi-center, prospective, mixed-methods study seeking to evaluate the clinical and cost-effectiveness of upgrading sound processors in older Australian adults with older technology was conducted alongside an Australian Government-funded CI upgrade program. The study included a diverse clinical cohort whose processors were obsolete and who did not have access to funding for replacement sound processors. Existing sound processors were replaced with the most current commercially available sound processor approved by the Therapeutic Goods Administration (TGA), specifically the Cochlear™ Nucleus^®^ 7 or Kanso^®^ 2 processors. These upgraded devices introduced two new technological features, as follows:

1. Forward focus. A noise reduction feature activated through the Smart App or a button push. 2. Connectivity. Direct, wireless streaming capabilities from any compatible smartphone via a phone clip [15].

The study was approved and conducted under the ethical oversight of the Human Research Ethics Committees of the St Vincent’s Hospital in Melbourne (HREC 039/21), Hearing Australia Human Research Ethics Committee (HAHREC/2020-28) in Sydney and the Royal Victorian Eye and Ear Hospital (HREC/10/978H/16) in Melbourne. Information about the study was provided to all participants and written and verbal informed consent was obtained from all participants.

The main inclusion criteria were being aged 65 years of age or older; having no access to private health insurance or other compensable funding stream that funds a replacement sound processor(s). The exclusion criteria were being unable to recall recent events due to age-related problems (e.g., cognitive decline or dementia). Recruitment for the study ran from June 2021 to May 2022 across 14 clinics around Australia (Amplify Hearing and Diagnostics (NSW), Clarity Hearing and Balance/QLD Vestibular (QLD), Cochlear Care Centre (VIC), Ear Science Institute (WA), Fiona Stanley Hospital (WA), Mater Health Services Brisbane Ltd. (QLD), Medical Audiology Services (WA), Royal Brisbane and Women’s Hospital (QLD), Royal Hobart Hospital (TAS), Royal Perth Hospital (WA), Royal Sir Charles Gairdner Hospital (WA), Royal Victorian Eye & Ear Hospital (VIC), South Australia Cochlear Implant Centre (SA), Townsville Hospital (North QLD CI Centre)).

### 2.2. Clinical Outcomes and Patient-Report Outcome Measures (PROMs)

**Figure 1:**
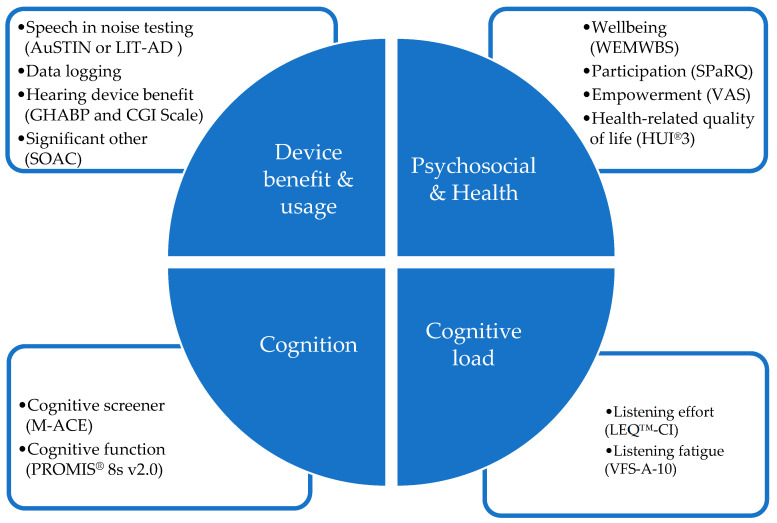
An overview of the four domains evaluated and clinical and patient-reported outcome measures used within each domain.

Figure 1 shows an overview of the four patient-centered core outcome domains evaluated and the measures used. Standard clinical measures were used alongside research aspects at 4 appointment intervals (pre upgrade appointment, upgrade appointment, 4 weeks post upgrade appointment and 6 months post upgrade appointment). The outcome measures used in each domain are show in Figure 1 below.

### 2.3. Study Regime and Protocol Scenarios

A decision tree chart was used to guide clinicians to determine the most appropriate outcome measure to accommodate a range of clients with diverse needs. The protocol scenario being applied to participants was based on participants who speak English at home and who could (Scenario 1) or could not (Scenario 2) attend a separate pre-upgrade appointment; culturally and linguistically diverse participants who could complete the study questionnaires with support (Scenario 3); and participants who could not complete the study tests/questionnaire but for whom the assessment of the upgrade was done by proxy via the participant’s communication partner (Scenario 4). Full details of the protocol scenario decision tree are provided in Appendix A. The four outcome domains were:

1. Device benefit and usage measures. Speech understanding was measured using the participant’s pre-upgrade sound processor and the new upgraded sound processor(s). The same CI electrical stimulation parameters, commonly referred to as the MAP, were retained after the upgrade and carried over from their previous sound processor. The decision tree chart was used to guide clinicians to determine the most appropriate speech measure to use for each (see Appendix A) to accommodate a range of clients with diverse needs. Three measures of speech understanding outcomes were incorporated to account for the different protocol scenarios. (1) An adaptive Australian Sentence Test in Noise (AuSTIN) test [16] was used to evaluate speech perception during noise for participants who spoke English as their first language (Protocol Scenarios 1 or 2 (Table A1 or Table A2)). The AuSTIN test comprises Bamford–Kowal–Bench-like open-set sentence lists presented under adaptive noise (four-talker babble). Speech reception thresholds (SRTs) were measured by presenting target speech from a loudspeaker directly in front (0° azimuth) of the participant and presented at a fixed level of 60 dB SPL. The uncorrelated four-talker babble noise was presented to the rear of the participant via a second loudspeaker (180° azimuth). The noise was varied in level (adaptively) according to the participants’ responses. The final outcomes measure was the mean signal-to-noise ratio score (SNR-50) in dB at which the participant registers 50% of the key words correctly. (2) The Language Independent Test of Auditory Discrimination (LIT-AD) [17] was used to evaluate participants who speak languages other than English (Protocol Scenario 3 (Table A3)). The LIT-AD test assesses a person’s ability to discriminate similar speech sounds. It consists of 81 speech sound sets in triplets (phonemes) equalized for discrimination difficulty and designed to be neutral across more than 40 languages. The phonemes are presented in noise shaped according to the international long-term speech spectrum [18]. (3) The Self-assessment of Communication–Significant Other (SOAC, 10Q) [19] self-report outcome measure was administered to the participants’ preferred communication partner to identify issues that hearing challenges may cause for significant others (Protocol Scenario 4 (Table A4)). Device usage via datalogging was obtained from the participant’s previous sound processor (if available) and from the upgraded sound processor for all participants, so as to measure device usage in various listening environments. Hearing device benefit (efficacy and effectiveness) was assessed using the Glasgow hearing-aid benefit profile (GHABP) [20].

2. Psychosocial and Health measures: The Warwick–Edinburgh Mental Well Being Scale (WEMWBS) (14Q) was administered to assess subjective well-being and psychological functioning [21]. The Social Participation Restrictions (SPaRQ-19Q) patient-reported outcome scale was used to assess participation and measure the impact of hearing loss on performing actions, thoughts and feelings experienced in a social context [22]. A visual analog scale (VAS) was used to obtain a participant’s overall subjective measure of empowerment. The Clinical Global Impression (CGI) scale was used to identify meaningful change for participants. The self-report scale includes 7 ratings ranging from ‘much worse’ to ‘much improved’. The scale allows for changes in the other outcomes to be related to whether the participant perceived a change, and if so, what size of change, and whether that change was positive or negative [23]. Health-related quality of life was assessed in terms of health utility, as measured using the Health Utilities Index Mark 3 (HUI3) [24]. 

3. Cognitive function measures: The Mini Addenbrooke’s Cognitive Exam (M-ACE) assessment tool [25] was used to screen cognitive function. The Patient-Reported Outcome Information System (PROMIS^®^ Adultv2.0 Cognitive Function-Short Form 8a) questionnaire was used to assess participant-perceived changes in cognitive functions including mental acuity, concentration, verbal and nonverbal memory, and verbal fluency [26]. 

4. Cognitive load measures: The Listening Effort Questionnaire–Cochlear Implant (LEQ-CI, 21Q) patient-reported outcome measure developed specifically to assess perceived listening effort in CI candidates and recipients [27], and The Vanderbilt Fatigue Scale for Adults with Hearing Loss (VFS-A-10) measure of listening-related fatigue [28], were used to measure cognitive load.

### 2.4. Qualitative Methodology Sub-Study

Regarding the qualitative interviews, data were collected through semi-structured interviews conducted remotely, with an interview guide that provided a general framework for the conversation, while permitting the participant to explore the effects of the upgrade on daily life, social participation, listening effort, and overall well-being on older adult participants with cochlear implants and their communication partner (please see Pang et al.’s paper for more detail). The interviews were audio-recorded and professionally transcribed verbatim by a secure third party, an online transcription service, after which two research team members manually de-identified all transcripts in preparation for data analyses.

### 2.5. Health Economic Evaluation of Cost-Effectiveness

For health economic evaluation, two state-transition microsimulation models of adults with one or two CIs were developed to predict outcomes over users’ lifetime, including sound processors or their parts failing or becoming faulty, future sound processor upgrades, and mortality [28,29,30,31,32]. The models tracked CI user age and sound processor age over a time horizon of up to 35 years. Inputs were based on the study, a survey of four clinics involved in the study, the published literature and administrative sources [33]. Costs were reported in 2022 AUD, and health outcomes were reported in terms of quality-adjusted life years (QALYs) [34]. The incremental cost-effectiveness ratio (ICER) was estimated, and extensive threshold, univariate, scenario and probabilistic sensitivity analyses were conducted [35,36,37,38].

### 2.6. Data Analysis

Data from speech assessments and self-report questionnaires were analyzed using descriptive statistics, and those statistics were used to generate visualizations and graphs of the data. Linear mixed models were fit to the data separately for each outcome, incorporating participant age, timepoint of data collection (pre- and then each post-upgrade timepoint), and study group as fixed factors and participant as a random factor. An interaction between timepoint and group was included in the model given that the size of the change between pre- and post-upgrade timepoints was expected to be larger in those using the oldest technology. Qualitative research analyses were conducted using the NVivo software (version 1.6.1, 2022) developed by Lumivero (Denver, CO, USA). The interview data were analyzed deductively using reflexive thematic analysis, a method outlined in Braun and Clarke [39,40,41]. The interview research participants were a subset of a larger cohort that completed the cochlear implant sound processor upgrade study, so in effect, this is a self-selecting sample, which is analogous to the purposive sampling technique employed for this study.

## 3. Results

The study start date was 20 May 2021, and it closed on 21 April 2023. Recruitment ran from June 2021 to May 2022; a total of 304 participants were recruited, distributed across the three participant groups, based on the sound processor technology worn (Table 1). These groups were Freedom or older processors (Group 1), Nucleus^®^ 5 processors (Group 2), and Nucleus^®^ 6 processors (Group 3). A total of 340 processors (296 Nucleus^®^ 7 and 44 Kanso^®^ 2) were allocated to participants with unilateral and bilateral CIs. At the commencement of the study, collaborating sites estimated the total number of patients that may use these obsolete or near-obsolete sound processors. Based on these estimates, the recruitment rates represent 78%, 77%, and 33% of the total population of these individuals who are managed by those sites across the three participant groups. Therefore, a significant majority of the entire clinical population of older adults using Nucleus^®^ 5 or an older technology (Groups 1 and 2) across the study sites was recruited into the study. Given that there was a far larger number of potential participants using Nucleus^®^ 6 technology (Group 3) compared to users of older technology (Groups 1 and 2), and that there was a limited quantity of sound processors available within the study budget, recruitment as a proportion of the population was lower in Group 3, but still sufficient to satisfy the project objectives. Recruitment into Group 3 was deliberately limited in order to ensure that the available processors were distributed across the three groups, rather than primarily to the largest group (Table 1).

**Figure 2:**
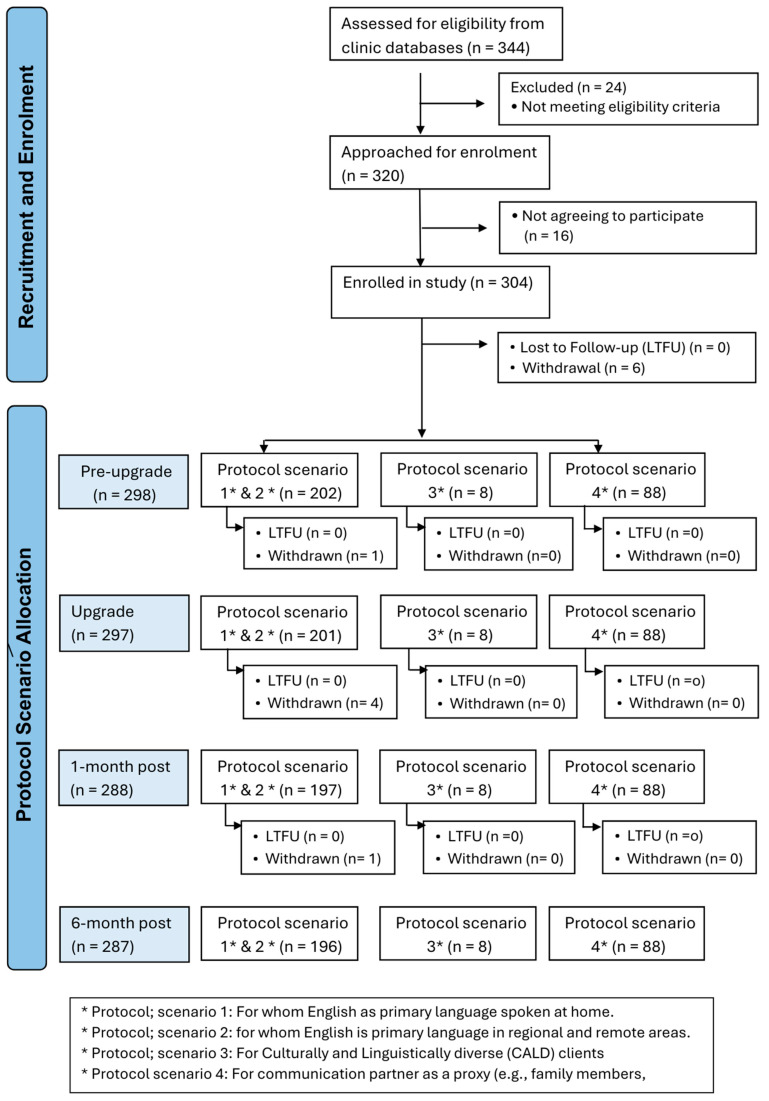
Shows the identification and enrolment flow diagram based on the protocol scenario being applied to participants.

An overview of the appointment schedule, data collection and participant numbers based on the participant protocol scenarios for all appointments is shown in Figure 2.

A majority of participants (84%) could speak English, and therefore fit into Scenario 1 or 2, with a majority of those fitting into Scenario 2 as COVID-19 restrictions limited the number of people who could attend an in-person pre-upgrade appointment. The far smaller numbers of participants following Scenarios 3 and 4 likely reflect the fact that they are a small proportion of the population of CI users at the participating sites, and this may ultimately limit the extent to which meaningful inferences can be drawn from the data provided by those groups. A total of 340 cochlear implant sound processors were upgraded in 304 adults. The majority of participants are users of a unilateral cochlear implant (n = 266), with a small proportion using cochlear implants bilaterally (n = 74). The cohort had a mean age of 77.4 years (SD 6.6); 147 (48.5%) were female and 157 (51.5%) were male. The age of onset of hearing loss was 30.0 (SD 21.0) years, and the duration of CI use was 12.0 (SD 6.2) years. The majority of participants were retired and reported living with others (Table 2).

### 3.1. Clinical Outcome Measures

**Figure 3:**
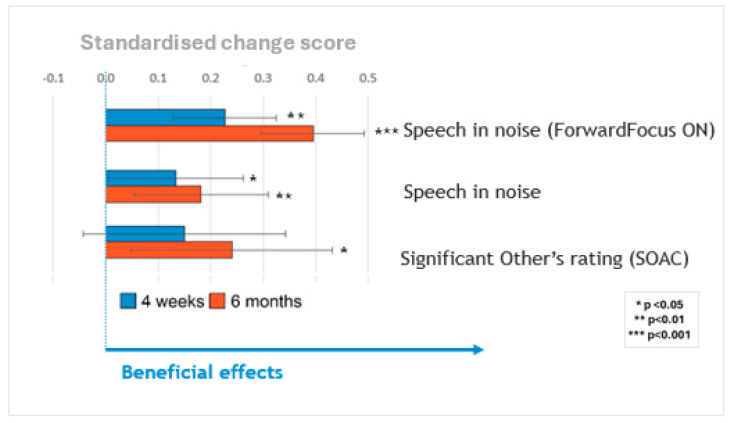
Primary outcome measures rescaled to a standardized change score. Effects are expressed in terms of standard deviations. A higher score indicates better performance for all speech understanding outcome measures.

The primary outcome is speech understanding during noise, which is the primary method used to assess the clinical effectiveness of the sound processor upgrades in participants who spoke English (Scenarios 1 and 2), who comprised the largest group assessed in the study. A major component of how newer sound processor technologies have improved outcomes is indicated by their ability to process and reduce background noise. The test used in this study was designed to evaluate how effectively these technologies assist individuals in noisy environments. The ForwardFocus technology in the upgraded processor is a user-activated feature aimed at enhancing face-to-face communication by attenuating noise from behind [15]. Figure 3 summarizes the main findings across these measures by plotting all primary measures on the same scale, and standardizing the change in scores at 4 weeks (blue bars) and 6 months (orange bars) relative to the pre-upgrade scores. Significant benefits were observed for speech in noise, with the ForwardFocus feature turned both on and off, and for reduced communication difficulties as perceived by significant others as a proxy. The participants’ ability to understand speech in noise improved significantly after the upgrade.

The largest benefits for speech perception were seen when the ForwardFocus feature of the Nucleus^®^ 7/Kanso^®^ 2 processors was enabled (4–8 dB improvement in speech reception thresholds), with benefits still present but considerably smaller when that feature was not enabled (~2 dB improvement).

Due to the very small number of participants completing the LIT-AD test, only the results of the AuSTIN test are presented here. Figure 3 summarizes changes in speech-in-noise scores at 4 weeks (blue bars) and 6 months (orange bars), relative to the pre-upgrade scores with ForwardFocus switched on and off. Participants’ abilities to understand speech in noise improved significantly after the upgrade. The greatest benefits to speech perception were observed when the ForwardFocus feature of the Nucleus^®^ 7/Kanso^®^ 2 processors was enabled, with benefits still present but considerably smaller when this feature was not enabled. All significant benefits were sustained throughout the 6-month follow-up period. The sizes of these benefits did not vary significantly based on the age of the sound processor(s) being upgraded, once the age of the participants was controlled for.

The self-assessment of communication partners, administered only to those following protocol scenario 4 (Table A4), showed a general pattern of reduced SOAC scores after the sound processor upgrade. This reduction in communication difficulties as perceived by significant others was statistically significant at 6 months, even after controlling for participant age. No effect of the study technology group was found.

**Figure 4:**
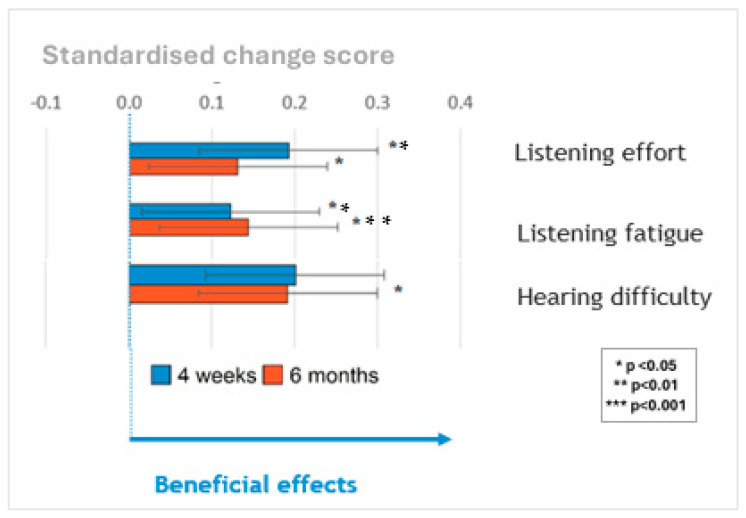
Secondary outcome measures rescaled to a standardized change score. Effects are expressed in terms of standard deviations. A higher score indicates better performance for all self-reported outcome measures.

A summary is shown in Figure 4 of performance in self-reported listening effort, listening fatigue and hearing difficulties measured on the same scale by standardizing the change in scores at 4 weeks (blue bars) and 6 months (orange bars) relative to the pre-upgrade scores. Upgrading sound processors significantly reduced listening effort and listening fatigue at 1 and 6 months and hearing difficulties at 6 months post-upgrade. The sizes of these benefits did not vary significantly based on the age of the sound processor(s) being upgraded once the age of the participants was controlled for.

No significant differences in other self-report measures (health utility, social participation, mental well-being, cognition and empowerment) were identified following the upgrade.

Data logging information captured by the sound processor device suggests that participants’ general usage of their processors was high and represented all-day use, or at least use for most of the participants’ waking hours. The inspection of the data from the environmental scene classifier incorporated into the study sound processors suggested that participants spent a majority of their time in quiet environments and made minimal use of the ForwardFocus feature (which has to be manually enabled on Nucleus^®^ 7/Kanso^®^ 2 processors), despite training on its use as part of the study protocol.

### 3.2. Qualitative Methodology

Twenty-two participants were interviewed for the qualitative sub-study, including seventeen adults who received the upgrade and five communication partners (typically family members, spouses, children, or friends). Participants represented all three technology groups and included those with one or two CIs from across Australian states. The findings were divided into pre-upgrade and post-upgrade experiences. Due to significant overlap in themes between CI users and their communication partners, the results were analyzed together, yielding 651 individual statements from the interviews, which were then grouped by shared meaning.

The sub-study revealed common experiences and concerns for both CI users and their communication partners. Participants reported that while older sound processors were better than hearing aids in noisy environments, they caused considerable stress if they failed, often impacting communication and well-being. Post-upgrade, improvements included enhanced social connections, better access to multimedia (e.g., music, podcasts, phone, and video calls), and improved hearing in noisy settings. These advancements were made possible by new features like Bluetooth connectivity and ForwardFocus in the upgraded processors. Communication partners noted that these improvements led to better social interactions, enhanced well-being, and a higher quality of life. For some older CI users, the upgrade was crucial to safety and a heightened awareness of their surroundings. Additionally, those who could not afford an upgrade felt relieved from concerns about their outdated processors failing—please see the full details in Pang et al. for more on this issue.

### 3.3. Cost-Effectiveness

A health economic analysis conducted from the perspective of the Australian Hearing Services Program, quantifying the health benefits of upgrades using the Health Utilities 3 questionnaire, found that upgrading sound processors is only cost-effective in terms of avoiding scenarios of total device failure, which is associated with a significant decrease in health-related quality of life, and ultimately justifies the cost of replacing devices when they fail. For unilateral CI users, if sound processors were beyond replacement or repair at 20 (15) years, then upgrading sound processors when they reach the age of around 11 years (4 years) would result in an incremental cost-effectiveness ratio for QALY (quality-adjusted life-year) (ICER) of AUD 50,000 per QALY gained. For bilateral CI users, if sound processors were beyond replacement or repair at 15 or 20 years, then upgrading sound processors when they reach the end of warranty (3 years) would result in an ICER of $50,000 per QALY gained. The ICERs decreased as the frequency of upgrading decreased, mainly due to the reduced cost of upgrading sound processors. Overall, the results were most sensitive to inutility due to sound processors ageing, if and when sound processors became beyond replacement or repair, and the time horizon of the analysis.

## 4. Discussion

The current report presents the findings from a multi-center study assessing a wide range of outcomes in adults over the age of 65 years in whom a cochlear implant sound processor was upgraded. These participants had varying generations of technologies in their existing sound processors. The overall results indicate that the clinical value of upgrading sound processors is evident when evaluation is conducted using both objective and subjective measures. However, the economic value primarily arises from protection against time ‘off air’ (i.e., no access to sound) that can occur when a processor fails. From the users’ perspective, upgrades reduce concerns about potential technology failures. This is significant because a sound processor failure means losing the ability to use the cochlear implant, thus limiting communication and engagement with the world around them.

The study examined a range of outcomes because the benefits of biomedical technology, such as cochlear implants, can be highly specific to an individual’s lifestyle, including their listening situations and activities most important to them. The most direct measure of changing sound processor technology was assessing speech perception in noise, which evaluated whether the newer technology enhances access to speech sounds. Even without the use of novel features such as ForwardFocus (which in the current study had to be manually enabled by the user in everyday situations, as in the Nucleus^®^ 7/Kanso^®^ 2 processors), benefits related to ability to understand speech and noise were observed. With access to ForwardFocus, even larger and more significant noise benefits were achieved. While the current study identified significant clinical benefits derived from upgrading sound processors for the individual, the sizes of those benefits did not differ statistically depending on the age of the sound processor technology that was being upgraded. Data logging information captured by the sound processor device suggests that participants spent a majority of their time in quiet environments, and made minimal use of the ForwardFocus feature (which has to be manually enabled on Nucleus^®^ 7/Kanso^®^ 2 processors) despite training on its use as part of the study protocol, which is perhaps to be expected given the demographics of the sample, the degree of their hearing loss, and the co-occurrence of COVID-related lockdowns.

The qualitative sub-study also highlights additional benefits not captured by self-report instruments. These include the benefits that can arise from access to more sophisticated connectivity technology in newer sound processors. Of particular note is the reassurance and sense of ease that the end users reported because they no longer had to worry about using a processor that may fail at an unpredictable point in time, where such a failure would have far-reaching effects on their ability to participate in many everyday activities.

The cost-effectiveness analysis, from the Australian health perspective, evaluated whether funding sound processor upgrades for adults over 65 is a cost-effective use of health care resources. Clinical feedback indicates that most failures are managed with like-for-like replacements. For obsolete processors, clinics use donated or unused devices to minimize ‘off air’ time. Due to the success in sourcing replacements and minimizing ‘off air’ time, regular upgrades were not deemed cost-effective for unilateral cochlear implants, as the added cost did not translate into additional health benefits, according to the HUI3 instrument used in the study.

It is uncertain if clinical sites can continue to obtain donations of old sound processors to keep patients with obsolete technology functional in the event of a total failure. Alternative scenarios were considered wherein processors become unrepairable and unreplaceable once out of manufacturer support, leading to severe hearing loss and decrements in health, reflecting their poorer hearing status resulting from an inability to use their CI. The cost-effectiveness of upgrades here depended on the assumed lifespan of devices (15 or 20 years) and upgrade frequency. The current study’s data could not predict the sustainability of the support model for obsolete technology, nor how the increased uptake of CIs might affect support for outdated devices. In those scenarios, the cost-effectiveness of upgrades was dependent on how long devices were assumed to last before ultimately being irreparable and unreplaceable (15 or 20 years), and how frequently upgrades were performed. It is not possible to use the data collected in the current study to make future projections regarding how sustainable the model of support is whereby clinical sites provide patients with obsolete technology. If the information on cost-effectiveness in this paper is used to inform health policy regarding sound processor upgrades, consideration should be given to whether the current model of supporting users of obsolete sound processor technology is sustainable, and therefore which of the modeled scenarios is most relevant to inform any decision-making process. Consideration should also be given to the fact that the current economic analysis had to rely on estimates for several key parameters. This was either because the required information was not publicly available and could not be obtained from the manufacturer (e.g., the reliability of processor models prior to the Nucleus© 7 and Kanso© 2), or because the information is not readily recorded by the clinics who manage these patients (e.g., how long a user is ‘off air’ due to a gradual or total failure of a sound processor). Finally, although the economic evidence alone for upgrading bilateral CI users’ processors appears to be cost-effective, consideration should be given to the relatively small sample upon which these results were based.

The decision to upgrade the sound processors of CI systems is predicated on the idea that benefits can be gained either from providing the end users with access to more up-to-date technology, or from minimizing the probability of failure and therefore the probability of adverse consequences for the individual related to becoming profoundly or severe-to-profoundly deaf, or both. The current results do suggest that in adults aged 65 years or older, upgrading sound processors can provide consistent clinical and more holistic benefits to the end user. However, perhaps surprisingly, the sizes of these benefits were similar even when the upgrade represented several generations of technological advancement (e.g., Freedom™ to Nucleus© 7). This pattern of results could have arisen because the newest technology that participants were upgraded to represents a step-change compared to all previous models, including the most recent generation of Nucleus© 6 processors, or perhaps because benefits arise from replacing any device that has degraded over a certain period of time.

The question of who should be responsible for supplying processor upgrades is inherently tied to broader issues of health equity, sustainability, and the evolving needs of cochlear implant (CI) users. As highlighted in our study, vulnerable groups, such as older adults in Australia, continue to face significant challenges in maintaining access to essential auditory technology. These challenges demonstrate that even in a country with universal health coverage provided to all Australian citizens and permanent residents, not all health services are fully covered, and disparities persist in the maintenance and upgrading of medical technologies.

In low- and middle-income countries (LMICs), where access to cochlear implants and subsequent upgrades is even more constrained, the roles of international aid organisations, governments, non-governmental organizations (NGOs) and manufacturers become crucial. These stakeholders might advocate for and help establish models that ensure CI users are not abandoned due to device obsolescence. As such, policy development and service delivery models must take into account the ongoing needs of individuals, ensuring that technology is maintained and updated in a sustainable way. Policies must address systemic barriers to access, ensuring upgrades are universally available, particularly for those at greater risk of marginalization, such as older adults and individuals in low- and middle-income countries.

If the information on cost-effectiveness presented in this paper is to inform health policy regarding sound processor upgrades, consideration should be given to the sustainability of the current model for supporting users of obsolete sound processor technology. It is also crucial to assess which of the modeled scenarios is most relevant for guiding decision-making in this context.

## 5. Strengths and Limitations

The research is not without its limitations. First, the current study was not a randomized controlled trial, resulting in the need to assume that the change in utility between baseline and follow-up was due to the upgrading of the sound processors. That said, given the age of the cohort, the likely onset of co-occurring health conditions, and the co-occurrence of the COVID-19 pandemic, it is likely that utilities would have decreased over this period, not increased. Second, HUI3 responses were missing for 3% of the respondents at baseline, 17% of the respondents at weeks 4 and 13% of the respondents at 6 months. A ‘last observation carried forward’ approach was used to minimize the impact. The age at which sound processors are beyond replacement or repair is unknown. Cochlear Ltd. currently sells each generation for around 10 years and provides support for an additional 5 years [42]. All participants had a working sound processor, with the maximum age of a sound processor being 19 years. This suggests the mean age beyond replacement or repair is between 15 and 20 years. As it is a key driver of the results, further research on this parameter is warranted. Finally, there were several potential benefits derivable from sound processor upgrades that were not captured in the model, including the impact on falls [43] and cognition [44], related to potential impacts on safety, mortality, and productivity. The approach taken in the cost-effectiveness analysis is conservative.

The analysis focused on sound processors made by Cochlear Ltd., as it has the highest market share in Australia. However, there are several other brands currently available in Australia. While the results may differ for other brands, sound processor costs are similar (Australian Government Department of Health and Aged Care, 2022 [45]); however, there is a lack of evidence regarding differences between cochlear implant systems produced by other manufacturers (National Institute for Health and Care Excellence, 2019 [46]). Nevertheless, it remains a fact that the smaller pool of CI users reduces the potential donation pool available to replace or repair older processors, which may reduce the age at which sound processors become beyond repair. Consequently, the results of this analysis should be generalizable to other CI systems.

## 6. Conclusions

This study represented an opportunity to conduct research with a large cohort of older Australians who use Cochlear™ cochlear implants (CIs), in order to examine the effects of upgrading sound processors. Overall, this study provides evidence that upgrading sound processors offers significant benefits for the individual, and is economically sound in cases where this process prevents someone losing access to sound completely due to a total device failure. The study has the potential to inform both Australian and international guidelines in terms of whether, for who, when, and at what cost sound processors should be upgraded in order to be cost-effective. In a broader context, this can inform a wider debate regarding the ongoing maintenance and accessibility of replacement devices for individuals lacking financial resources, as the rising prevalence of medically implanted devices poses a significant public health challenge.

## Figures and Tables

**Table 1:** Study technology groups. Estimated total number of eligible adults with outdated technology identified by study sites and the number of those adults recruited.

Study Technology Group	Total Number of Eligible Adults Estimated by Sites	Number of Eligible Adults Targeted for Recruitment	Number Recruited
Freedom™ or older	89	89	69
2. Nucleus® 5	151	151	117
3. Nucleus® 6	360	104	118
Totals	600	344	304

**Table 2:** Participant demographics.

All				Freedom		Nucleus 5		Nucleus 6	
Demographics		n	%	n	%	n	%	n	%
Gender									
	Male	103	51.5	20	52.60	39	52.70	44	50.00
	Female	97	48.5	18	47.40	35	47.30	44	50.00
Cause of hearing loss									
	Unknown	58	29.0	11	28.90	18	24.30	29	33.00
	Two causes selected	34	17.0	8	21.10	12	16.20	14	15.90
	Other	31	15.5	7	18.40	11	14.90	13	14.80
	Noise exposure	21	10.5	3	7.89	9	12.20	9	10.20
	Genetic	23	11.5	3	7.89	8	10.80	12	13.60
	Illness	9	4.5	2	5.26	4	5.41	3	3.41
	Three causes selected	8	4.0	1	2.63	4	5.41	3	3.41
	No cause selected	6	3.0			5	6.76	1	1.14
	Injury or accident	4	2.0	1	2.63	2	2.70	1	1.14
	Ossicular condition	2	1.0	1	2.63	1	1.35		
	Four or more causes selected	3	1.5					3	3.41
	Syndrome	1	0.5	1	2.63				
Tinnitus									
	Yes	105	52.5	27	71.10	31	41.90	47	53.40
	No	90	45.0	11	28.90	38	51.40	41	46.60
	(Left Blank)	5	2.5			5	6.76		
Listening Difficulties									
	A little	77	38.5	13	34.20	30	40.50	34	38.60
	Often	47	23.5	13	34.20	13	17.60	21	23.90
	About half	36	18.0	7	18.40	11	14.90	18	20.50
	Always	26	13.0	4	10.50	13	17.60	9	10.20
	Non at all	9	4.5	1	2.63	2	2.70	6	6.82
	(Left Blank)	5	2.5			5	6.76		
Current occupation									
	Retired	182	91.0	33	86.80	67	90.50	82	93.20
	Working part time	5	2.5	1	2.63			4	4.55
	Marked_N/A	5	2.5	3	7.89	1	1.35	1	1.14
	(Left Blank)	5	2.5			5	6.76		
	Working full time	3	1.5	1	2.63	1	1.35	1	1.14
Education Level									
	7 to 12 years of schooling	130	65.0	28	73.70	45	60.80	57	64.80
	Diploma or Certificate	33	16.5	2	5.26	14	18.90	17	19.30
	University qualification	16	8.0	5	13.20	3	4.05	8	9.09
	6 or less schooling	15	7.5	3	7.89	6	8.11	6	6.82
	(Left Blank)	6	3.0			6	8.11		
Living Situation									
	Lives with others	111	55.5	23	60.50	36	48.60	52	59.10
	Lives alone	69	34.5	12	31.60	28	37.80	29	33.00
	Lives alone (with carer support)	15	7.5	3	7.89	5	6.76	7	7.95

**Figure A1:**
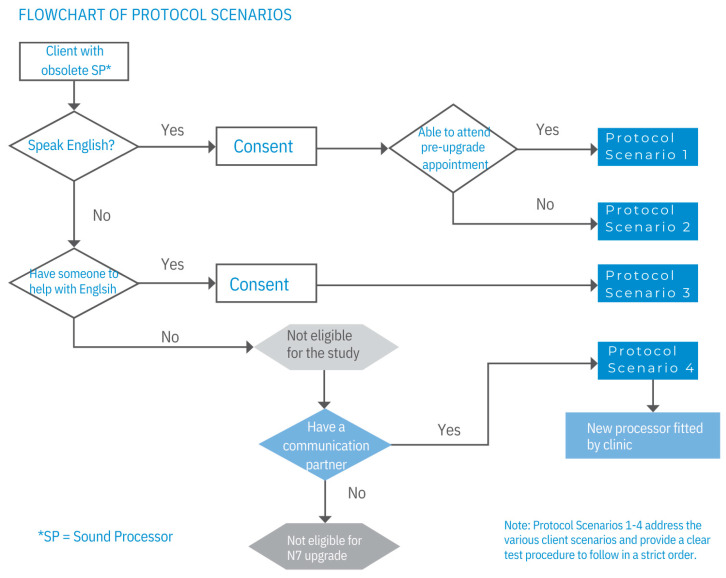
Flowchart of Participant Protocol Scenarios.

**Figure A2:**
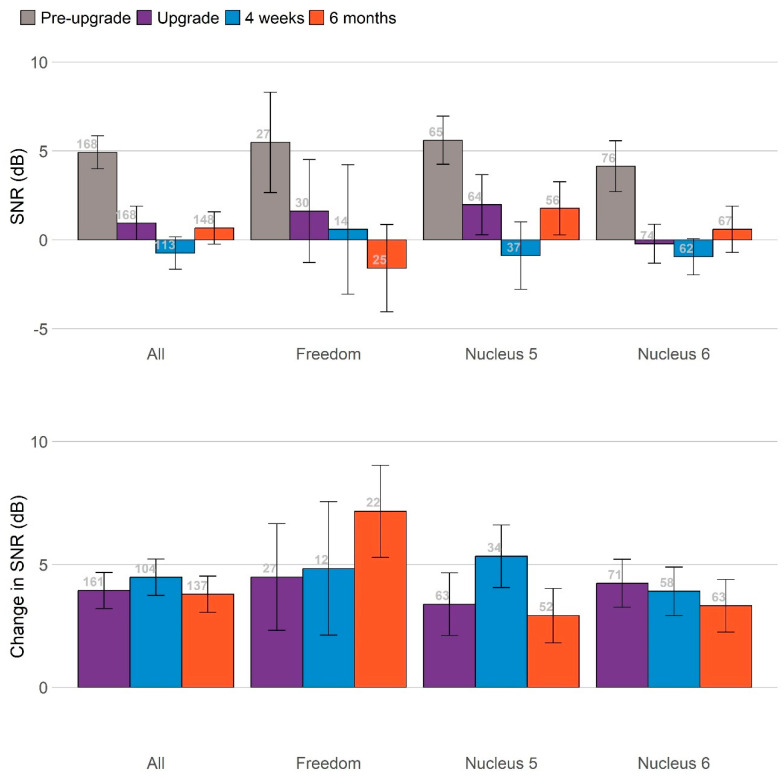
ForwardFocus on: (**Top panel**). Mean SRT obtained with ForwardFocus technology switched off (SRT FF off) at pre-upgrade, and mean SRT obtained with ForwardFocus switched on (SRT FF on) at upgrade, 4 weeks, and 6 months. (**Bottom panel**). Mean change in SRT obtained with ForwardFocus technology switched off (SRT FF off) at pre-upgrade vs. upgrade, 4 weeks, and 6 months with ForwardFocus switched-on.

**Figure A3:**
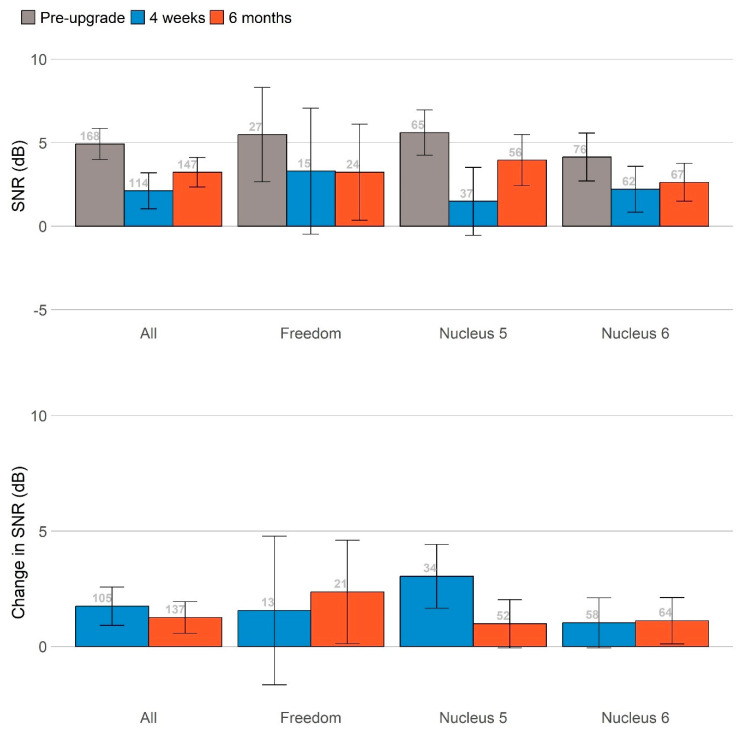
ForwardFocus off: (**Top Panel**). Mean SRT obtained with ForwardFocus technology switched off (SRT FF off) at pre-upgrade, 4 weeks, and 6 months. (**Bottom panel**). Mean change in SRT obtained with ForwardFocus technology switched off (SRT FF off) at pre-upgrade vs. 4 weeks, and 6 months.

**Figure A4:**
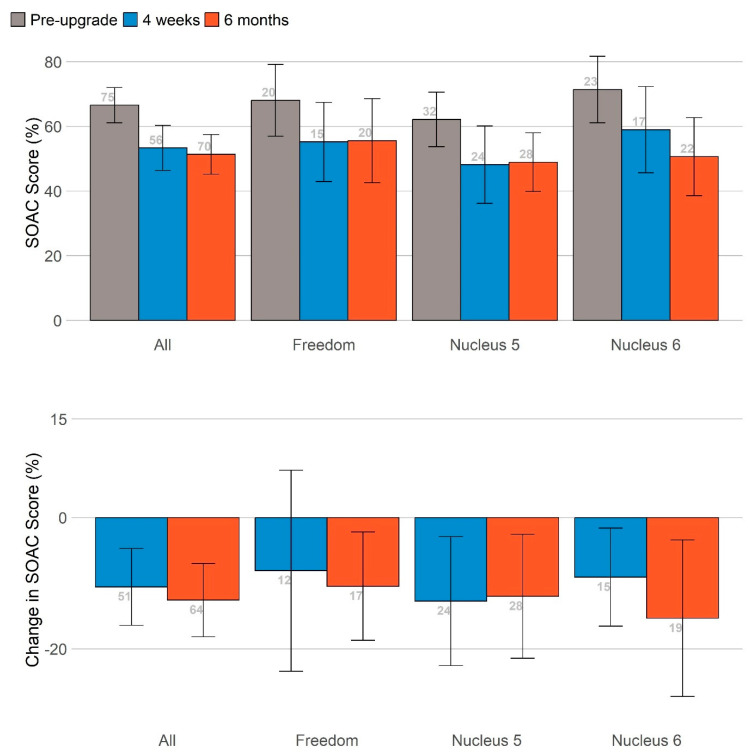
SOAC assessment: (**Top Panel**). Mean SOAC scores (%) at pre-upgrade, 4 weeks, and 6 months for participants in the communication community study arm. (**Bottom Panel**). Mean change in SOAC scores at pre-upgrade vs. 4 weeks and 6 months.

**Figure A5:**
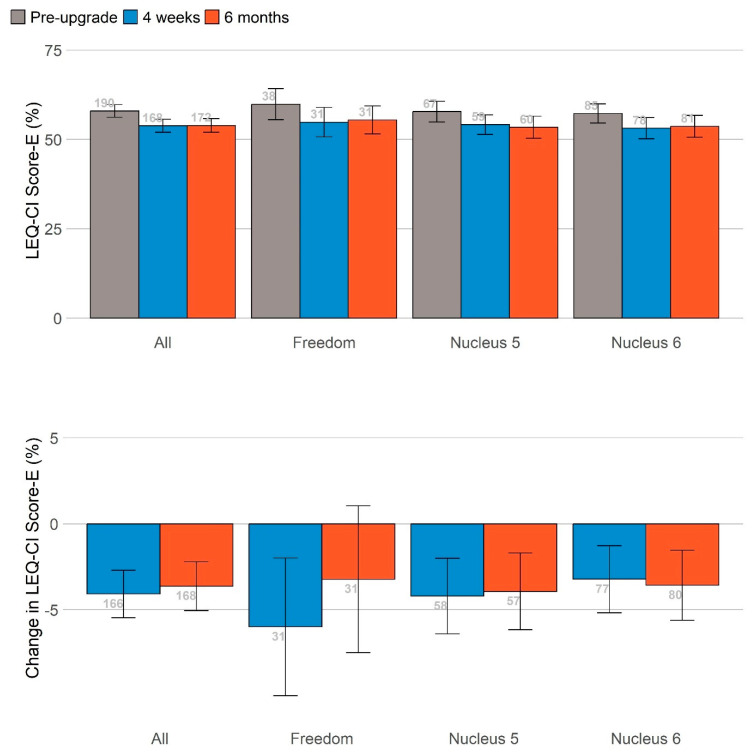
LEQ-CI assessment: (**Top panel**). LEQ-CI Scores at pre-upgrade, 4 weeks, and 6 months. (**Bottom panel**)**.** LEQ-CI scores from pre-upgrade to 4 weeks and 6 months.

**Figure A6:**
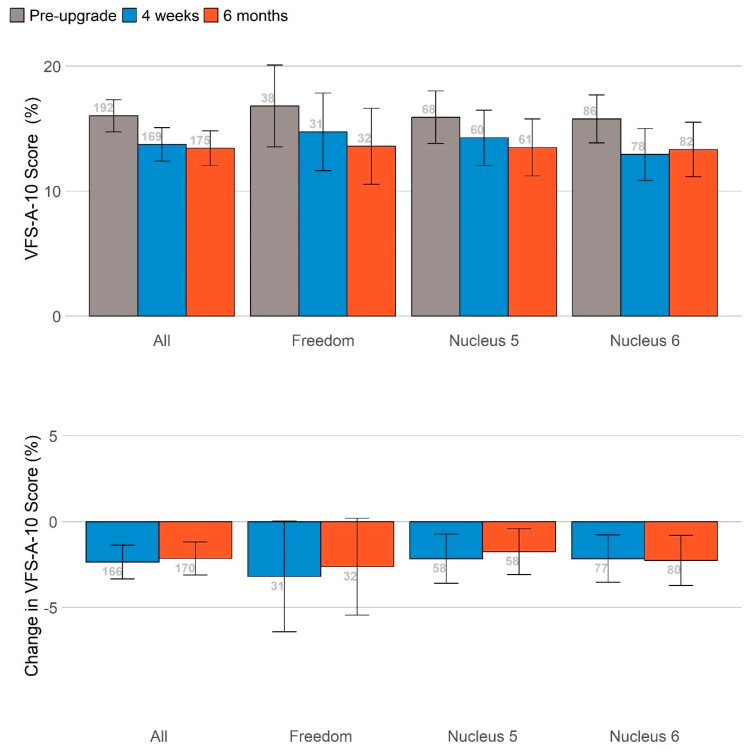
VFS-A-10 assessment: (**Top panel**). VFS-A-10 (Fatigue) scores at pre-upgrade, 4 weeks, and 6 months. (**Bottom panel**)**.** VFS-A-10 (Fatigue) scores from pre-upgrade to 4 weeks and 6 months.

## Data Availability

Any request for access to anonymized data should be directed to paola.incerti@nal.gov.au.

## Appendix A

**Table A1:** Protocol Scenario 1: Standard procedure for clients who speak English.

Measures	Pre- Upgrade	Upgrade	+4 Weeks Post	+6 M Post
Demographic Q (incl. Digital literacy)	or			
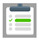
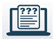
2. M-ACE memory test				
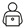
3. AuSTIN speech test				
4. Check Datalogging				
5. Fitting new processor and instructions				
6. CGI rating scale			or	or
7. PROMIS^®^ Q	or		or	or
8. HUI-3 Q	or			or
9. GHABP Q	or		or	or
10. SPaRQ Q	or		or	or
11. LEQ_CI Q	or		or	or
12. WEMWBS Q	or		or	or
13. Empowerment VAS	or		or	or
14. Check questionnaires (if paper version) are completed before client leaves				

At the beginning of each questionnaire, there are options to identify if the form is completed by clients or by helpers (e.g., family member/clinician/interpreter). Note:  direct measure or check or  paper questionnaire or  online questionnaire.

**Table A2:** Protocol Scenario 2: Procedure for clients who speak English but cannot attend the pre-upgrade appointment at the CI center (including clients in regional and remote areas or due to COVID-19 restrictions).

Measures	Pre- Upgrade	Upgrade	+4 Weeks Post	+6 M Post
Demographic Q (incl. Digital literacy)				
2. M-ACE memory test with previous sound processor				
3. AuSTIN speech test with previous sound processor				
4. Check Datalogging with previous sound processor				
5. Fitting new processor and instructions			Check datalogging with new sp	Check datalogging with new sp
6. AuSTIN speech test with new processor				
7. M-ACE withnew processor				
8. CGI rating scale			or	or
9. PROMIS^®^ Q			or	or
10. HUI-3 Q				or
11. GHABP Q			or	or
12. SPaRQ Q			or	or
13. LEQ_CI Q			or	or
14. WEMWBS Q			or	or
15. Empowerment VAS			or	or
16. Check questionnaires (if paper version) are completed before client leaves				

At the beginning of each questionnaire, there are options to identify if the form is completed by clients or by helpers (e.g., family member/clinician/interpreter). Note:  direct measure or check or  paper questionnaire or  online questionnaire

**Table A3:** Protocol Scenario 3: Procedure for culturally and linguistically diverse clients that have a translator or preferred communication partner to assist with questionnaires.

Measures	Pre- Upgrade	Upgrade	+4 Weeks Post	+6 M Post
Demographic Q (incl. Digital literacy)	or			
2. Check Datalogging				
3. Fitting new processor and instructions				
4. LIT speech test				
5. CGI rating scale			or	or
6. PROMIS^®^ Q	or			or
7. HUI-3 Q	or			or
8. GHABP Q	or		or	or
9. SPaRQ Q	or		or	or
10. LEQ_CI Q	or		or	or
11. WEMWBS Q	or		or	or
12. Empowerment VAS	or		or	or
13. Information and consent form with communication partner	or			
14. Self-assessment of Communication-Significant Other (SOAC, 10Q) by communication partner	or	or	or	or
15. Check questionnaires (if paper version) are completed before client leaves				

Note:  direct measure or check or  paper questionnaire or  online questionnaire.

**Table A4:** Protocol Scenario 4: Procedure for clients who do not have the capacity to consent to the study but wear an obsolete sound processer (i.e., Freedom, ESPrit, Sprint) and have a member of their communication community present to assist.

Measures	Pre- Upgrade	Upgrade	+4 Weeks Post	+6 M Post
Information and consent form with communication partner	or			
2. Self-assessment of Communication-Significant Other (SOAC, 10Q) by communication partner	or	or	or	or

Note:  paper questionnaire or  online questionnaire.

## Appendix B. Study Technology Group Primary Outcomes

### Appendix B.1. Speech Understanding

A summary of performance in the speech in noise test at the three timepoints with an upgraded sound processor (specific technology—ForwardFocus) switched on post-upgrade is shown in Figure A2, and the same for ForwardFocus switched off post-upgrade is shown in Figure A3. A more negative (lower) dB value indicates better performance in this test. Significant reductions in speech thresholds, representing significant improvements in performance, were identified at all timepoints under both testing conditions. No effect of study technology group was found once age had been accounted for.

### Appendix B.2. The Self-Assessment of Communication Community Partner

A summary of the self-assessment performed by the communication community partner (SOAC) of the upgrade performed with the study technology at three timepoints is shown in Figure A4 A lower score indicates better performance in this assessment, which was administered only to those following protocol scenario 4 (see Table A4 in Appendix A). The general pattern of results shows a reduction in the SOAC score following the sound processor upgrade, which was statistically significant at 6 months even when the age of the participant was controlled for. No effect of study group was found once age was accounted for.

### Appendix B.3. Self-Reported Listening Effort and Fatigue

The administration of the LEQ-CI and VFS-A-10 questionnaires identified reductions in both listening effort and fatigue, as shown by reductions in the effort scores (Figure A5) and fatigue scores (Figure A6) following the sound processor upgrade. Both effects were confirmed to be large and statistically significant, with no differences identified across study technology groups.
